# Bryozoan diversity on a whale bone: an uncommon substrate from the continental shelf off NW Spain

**DOI:** 10.1007/s12526-021-01189-6

**Published:** 2021-05-14

**Authors:** Javier Souto, Oscar Reverter-Gil

**Affiliations:** 1 Institut für Paläontologie, Geozentrum, Universität Wien, 1090 Vienna, Austria; 2 Museo de Historia Natural da Universidade de Santiago de Compostela, Parque Vista Alegre s/n, 15705 Santiago de Compostela, Spain

**Keywords:** Whale-falls, Colonization, Biodiversity, *Escharella*

## Abstract

Carcasses and bones of whales are interesting yet poorly understood habitats for invertebrates and microbial communities. Nevertheless, bryozoans have never before been reported in this particular habitat at the present day. Here, we present the first study on a bryozoan community colonizing a whale bone from the continental shelf off NW Spain at 128 m depth. Thirty-three species of bryozoans were identified, representing 50% of the known bryozoan diversity from this area and depth range. Despite this high biodiversity, no potential whale-fall specialists were detected: all the species are already known in the area, with several being more frequent in shallower or deeper waters. Our results support the hypothesis that the number of specialist taxa on whale-fall habitats decreases drastically on whale remains at depths of less than 260 m. These results also support the theory that whale-falls can serve as intermediate habitats where no other suitable substrate is present, facilitating habitat colonization.

**Supplementary Information:**

The online version contains supplementary material available at 10.1007/s12526-021-01189-6.

## Introduction

Carcasses and bones of whales, known as whale-falls, have been reported as interesting but poorly understood habitats, mainly for invertebrates and microbial communities. Previous studies have focused on different aspects of the biology, ecology and taxonomy of the invertebrate faunas and communities associated with natural and experimentally introduced whale carcasses. Such communities have been studied in different parts of the world at a wide range of depths, from a few meters down to about 3000 m depth (Smith and Baco 2003; Smith 2006; Braby et al. 2007; Lundsten et al. 2010a, 2010b; Glover et al. 2013; Smith et al. 2015; Taboada et al. 2016).

When whale carcasses sink, four overlapping stages of ecological succession have been defined (Smith et al. 1989, 2015; Bennett et al. 1994; Smith and Baco 2003): a mobile-scavenger stage, when soft tissues are removed by dense aggregations of necrophages; an enrichment-opportunistic stage, when dense assemblages of opportunistic invertebrates, especially polychaetes and crustaceans, colonize bones and the organically enriched sediments surrounding the whale-fall; a sulphophilic stage, during which bones are colonized by chemoautotrophic sulphur-based assemblages and emit sulphide during whale tissue decay; and a reef stage, after the depletion of organic matter retained in the bones, colonized primarily by suspension feeders. During the sulpholithic stage, which can last for more than 50 years, certain polychaete suspension feeders such as sabellids, chaetopterids and serpulids start to colonize the bones (Baco-Taylor 2002; Smith and Baco 2003). Moreover, during the last two stages, when scavengers and opportunistic species have consumed the flesh from carcasses, whale bones can sustain invertebrate communities for years thanks to the slow degradation rate of the components that form the bones (Smith and Baco 2003).

Major whale-fall reviews were published by Smith and Baco (2003) and Smith et al. (2015), including results about biodiversity, ecology and evolution. The latter authors listed 99 species associated with whale-falls, including Polychaeta, Aplacophora, Arthropoda, Cnidaria, Cephalocordata, Mollusca and Sipunculida, some of them showing unusual adaptations to deep-sea whale-fall habitats. Interestingly, the studies on these special and ephemeral habitats yielded no bryozoan records at the present day; only Boessenecker and Fordyce (2015) report data of the presence of fossil bryozoans in an also fossil whale bone. Smith et al. (2015) do, however, indicate that we are still in the early stages of discovery of the deep-sea whale-fall fauna.

Bryozoans are colonial suspension feeders that live attached to hard or ephemeral substrates, or are rooted within loose sediment (McKinney and Jackson 1989). About 6000 species have been described, although the estimated total number is 11,000 (Appeltans et al. 2012). Most bryozoans brood their embryos in specialized brooding cavities (ovicells or gonozooids) and release non-planktotrophic trochophore larvae; these spend only minutes to a few hours in the water column before settlement, metamorphosis and foundation of a new colony (Reverter-Gil et al. 2016).

In the present study, a whale bone was collected off the NW of the Iberian Peninsula. It was covered by many bryozoan colonies. The species have already been reported in several taxonomic papers (Reverter-Gil and Fernández-Pulpeiro 1999a, 1999b, 2001, 2007; Reverter-Gil et al. 2019a, 2019b) (Table 1). The bryozoan fauna of the Iberian Peninsula as a whole is well known, although the studies are irregularly distributed, both geographically and bathymetrically. The Iberian Peninsula hosts about 540 species, of which ca. 420 are present on the Atlantic coast (Reverter-Gil et al. 2016 and unpublished data). The bryozoan fauna from the present study area (Galicia, NW Spain) is the best known, by far, of the entire Iberian Peninsula, with a wealth of publications from the late nineteenth century until now, recording bryozoans from shallow waters down to about 2000 m depth. Altogether, these references include 280 species of bryozoans from Galicia (Reverter-Gil and Souto 2017; Reverter-Gil et al. 2019a, 2019b and unpublished data).

**Table 1:** Bryozoan species present between 100 and 200 m depth in Galicia. Species identified on the whale bone are marked with an asterisk (*)

	Depth range in the area (m)
*Aetea longicollis	128
*Alderina imbellis	9–550
*Amphiblestrum flemingii*	25–1094
*Arthropoma cecilii	0–300
*Bryocryptella torquata*	185–300
*Buffonellaria muriella*	172–186
*Callopora dumerilii	0–249
*Cellaria fistulosa*	0–186
*Cellaria salicornioides*	133
*Cellaria sinuosa*	17–119
*Celleporina derungsi*	40–119
*Celleporina hassallii*	0–172
*Chaperiopsis annulus	9–186
*Cheiloporina circumcincta	100–249
*Chorizopora brongniartii	0–249
*Collarina gautieri*	8–172
*Cribrilaria arrecta	20–172
*Cribrilaria innominata*	8–249
*Cribrilaria venusta	45–249
*Dentiporella saldanhai*	143
*Diplosolen obelium	16–249
*Disporella* sp.	100–150
*Entalophoroecia deflexa	10–850
*Entalophoroecia robusta*	130–145
*Escharella ventricosa	12–128
*Escharina vulgaris	15–190
*Escharoides coccinea*	0–186
*Fenestrulina asturiasensis	95–220
*Fenestrulina malusii*	0–249
*Figularia figularis	9–223
*Haplopoma sciaphilum	128
*Hemicyclopora discrepans	128–760
*Herentia sp.	128
*Hippoporina pertusa*	9–172
*Hippothoa flagellum	0–654
*Micropora normani*	16–249
*Microporella ciliata	0–249
*Omalosecosa ramulosa*	10–186
*Palmiskenea skenei*	117–530
*Pentapora foliacea*	0–249
*Plagioecia patina*	9–550
*Plagioecia sarniensis	9–300
*Porella compressa*	16–300
*Prenantia cheilostoma*	8–172
*Reptadeonella insidiosa	15–128
*Reteporella beaniana*	14–530
*Reteporella couchii	94–300
*Rhynchozoon bispinosum	0–190
*Schizomavella auriculata	4–249
*Schizomavella cornuta*	0–147
*Schizomavella discoidea	14–128
*Schizomavella hondti*	11–172
*Schizomavella linearis	0–1094
*Schizomavella teresae*	3–125
*Schizoporella cornualis	19–249
*Schizotheca fissa*	3–249
*Schizotheca tuberigera	128
*Scrupocellaria inermis	16–128
*Smittina cervicornis*	23–392
*Smittina landsborovii*	0–166
*Smittoidea reticulata	45–249
*Stephanollona armata	119–190
*Steraechmella buski	128–249
*Tervia irregularis*	185–990
*Tubulipora liliacea*	0–166
*Turbicellepora cantabra*	14–186

Here we study for the first time the bryozoan community associated with a whale bone, and compare this diversity with previous data from the same area and depth range.

## Material and methods

**Fig. 1:**
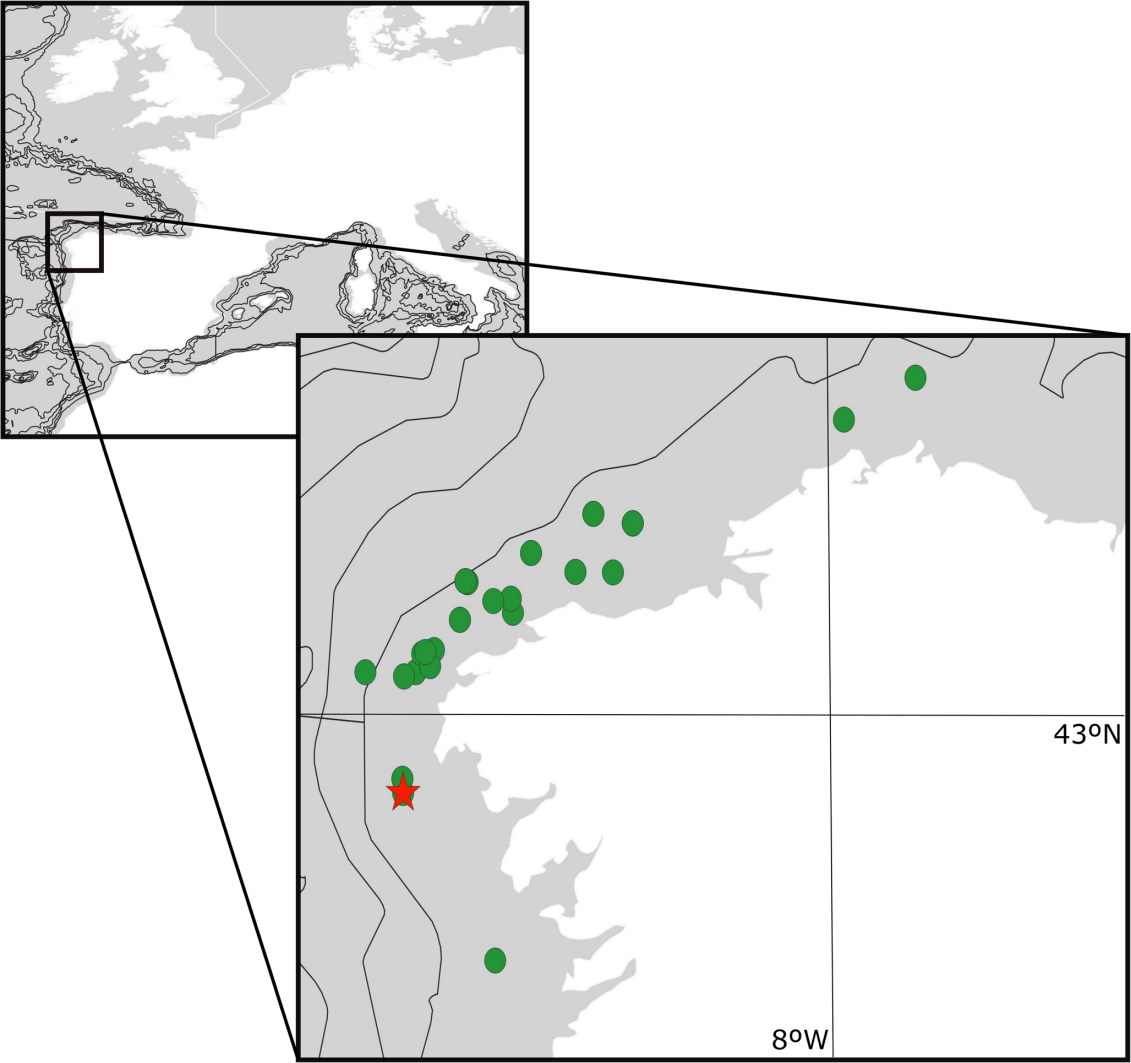
Map of the Northwest Iberian Peninsula with the locality where the whale rib was collected (red star) and localities between 100 and 200 m depth used for comparison (green circle)

During bottom-trawling fishing activities, a whale rib about two meters long by about 10 cm wide (i.e., ~ 0.2 m^2^) was collected on the continental shelf off NW Spain (42.80833° N, 09.39500° W, 128 m depth, May 1997) (Fig. 1). This area is a sedimentary plain. The sediments comprise mixed siliciclastic carbonate sands and muds, with small grain sizes that decrease offshore (Rey et al. 2014). The bone was delivered to the Department of Zoology of the University of Santiago de Compostela, where the presence of encrusting bryozoans was detected.

Bryozoan colonies were sorted by cutting the bone and studied with a stereo microscope. For definitive identification, selected specimens were cleaned with water and bleached for study using scanning electron microscopy (SEM). LEO 435 VP and Zeiss EVO LS15 SEMs were used at the University of Santiago de Compostela, and FEI Inspect S50 at the University of Vienna, always with a back-scattered electron detector in low variable vacuum mode.

To evaluate the importance of the bryodiversity of this substrate, the data were compared with the known records from the NW of the Iberian Peninsula (Galicia) extracted from our own Iberian bryozoan database. This database includes records from the literature as well as unpublished data. For comparative purposes, we have limited the data to species present in the range between 100 and 200 m depth. This area, with a few local exceptions, is a very homogeneous oceanographic area with the same currents affecting the entire continental margin (Barton 2001; Bower et al. 2002). The main substrates previously known for each species were extracted from the literature.

Most of the specimens studied from the whale bone are now stored in the Museo de Historia Natural da Universidade de Santiago de Compostela (MHNUSC-Bry), Spain (Supplementary Table S1).

## Results

Thirty-three species of bryozoans were identified growing on the whale bone (Table 1) (for taxonomic remarks, see also Reverter-Gil and Fernández-Pulpeiro 1999a, 1999b, 2001, 2007; Reverter-Gil et al. 2019a, 2019b). For 15 of these species, the results show an extension of the known depth range in the area. Previous data from 21 localities close to the whale bone locality and with similar depths (100–200 m) encompass a total of 51 bryozoan species (Fig. 1, Table 1).

Most of the species we found on the whale bone are common in the Atlantic waters of the Iberian Peninsula, with previous records in nearby areas, but have never been collected before on this special substrate. Nonetheless, the material also contained some poorly known species. For example, *Aetea longicollis* Jullien in Jullien & Calvet, 1903, was described originally from the north of Spain at 134 m and was collected thereafter at only two localities between 201 and 549 m depth in the north of the Bay of Biscay (Hayward and Ryland 1978). Little information is available about its substrate, but the species apparently prefers biogenic carbonate such as shells and corals (Jullien and Calvet 1903; Hayward and Ryland 1978). *Fenestrulina asturiasensis* Álvarez, 1992c is another poorly known species, described from the north of Spain at 120 m depth on a brachiopod shell. It was only recently reported from this whale bone by Reverter-Gil et al. (2019a), who also figured the species using SEM for the first time.

Although most of the species have already been recorded at similar depths, some are considered to be more common in shallower waters. These include *Callopora dumerilii* (Audouin, 1826), *Chorizopora brogniartii* (Audouin, 1826), *Figularia figularis* (Johnston, 1847), *Microporella ciliata* (Pallas, 1766), *Plagioecia sarniensis* (Norman, 1864), *Rhynchozoon bispinosum* (Johnston, 1847), *Schizomavella* (*Calvetomavella*) *discoidea* (Busk, 1859) and *Schizomavella* (*Schizomavella*) *auriculata* (Hassall, 1842) (see, e.g. Álvarez 1987; Reverter-Gil and Fernández-Pulpeiro 2001; Reverter-Gil et al. 2014).

Two other species, *Chaperiopsis annulus* (Manzoni, 1870) and *Schizoporella cornualis* Hayward & Ryland, 1995, were previously collected only in shallower waters in NW Spain (Reverter-Gil and Fernández-Pulpeiro 2001), but they were recently also collected in deeper waters (down to 249 m depth) (Reverter-Gil et al. 2019b) in the same area.

*Haplopoma sciaphilum* Silén & Harmelin, 1976, has a more distinctive habitat, namely, on stones in dark locations and caves down to 40 m depth (Silén and Harmelin 1976; Boury-Esnault et al. 2001), and also on small stones in deeper waters (Hayward and Ryland 1999).

In contrast, some species are more typical of deeper waters in the same area: *Hemicyclopora discrepans* (Jullien & Calvet, 1903) was previously recorded in Galicia only below 695 m, although it had been recorded by Jullien and Calvet (1903) in one locality in the nearby Bay of Biscay at 134 m depth. *Cheiloporina circumcincta* (Neviani, 1896) and *Steraechmella buski* Lagaaij, 1952, were found in the area down to 249 m depth, yet occur even down to 760 m depth in other Iberian Atlantic localities (Harmelin and d’Hondt 1992; Álvarez 1992a, 1992b).

Reverter-Gil and Fernández-Pulpeiro (2001) reported several ovicelled colonies of *Herentia hyndmanni* (Johnston, 1847) that were collected on this whale bone. This species has been recently redescribed by Berning et al. (2008), who establish the characters to differentiate it from the very similar species *Herentia thalassae* David & Pouyet, 1978, and indicate the difficulty in confirming the previous identifications without examining the original material. Both species are present in NW Spain (Reverter-Gil et al. 2019a). Unfortunately, the original material reported as *H. hyndmanni* is lost, and we have been unable to find more colonies on the whale bone. Accordingly, we correct here the previous identification as *Herentia* sp. Both species of *Herentia* were recorded at similar depths, although *H. hyndmanni* seems to have a wider bathymetrical distribution (Berning et al. 2008).

**Fig. 2:**
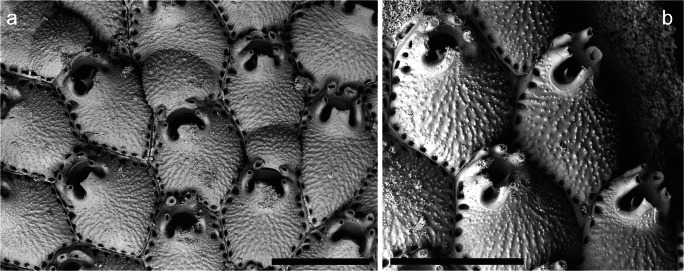
*Escharella ventricosa* on the whale bone, a ovicelled and non-ovicelled zooids in a colony (MHNUSC-Bry 415); **b** several autozooids showing lyrula whose lateral corners project like curved spines (MHNUSC-Bry 411). Scale bars: 0.5 mm

Finally, the specimens identified as *Escharella ventricosa* (Hassall, 1842) (Fig. 2) feature a conspicuous development of the lateral corners of the lyrula; these corners project laterally like curved pointed spines, which has not been observed previously in this species. Nevertheless, the other characters of the specimens are identical to the descriptions given in the literature for this species (e.g. Hayward and Ryland 1999). This character may reflect ecomorphological variability. This species was not previously recorded in Galicia below 36 m depth, but was recorded at 240 m in the nearby Bay of Biscay (Jullien and Calvet 1903).

## Discussion

Whale bone habitats share few species with deep-sea hard substrata, containing a high number of potential whale-fall specialists (Smith and Baco 2003). Nevertheless, the number of specialists on whale-fall habitats decreases drastically on whale remains at depths of less than 260 m (Smith et al. 2015). The present study did not detect any potential whale-fall specialists among the bryozoans on the whale bone. All the species are already recorded in the area, although some are poorly known or prefer a different depth range. Thus, some have been reported from deeper waters (e.g. *Cheiloporina circumcincta*, *Hemicyclopora discrepans* and *Steraechmella buski*), whereas others usually prefer shallower waters, reaching the photic zone, among them *Callopora dumerilii*, *Chorizopora brogniartii*, *Figularia figularis*, *Microporella ciliata*, *Plagioecia sarniensis*, *Rhynchozoon bispinosum*, *Schizomavella auriculata* and *Schizomavella* (*Schizomavella*) *linearis* (Hassall, 1841). Note, however, that recent studies (Reverter-Gil et al. 2019a, 2019b) indicate that some species previously considered more typical of shallow waters in NW Spain could be more widely distributed in deeper waters. Such bottoms have been little studied because they are mainly soft sediment habitats, which are less favourable for most bryozoans species.

An interesting feature of whale-fall habitats is that they appear to harbour the highest diversity of any deep-sea hard substrate (Smith and Baco 2003; Smith et al. 2015). This is reflected in the high bryozoan diversity we found on this single bone. Omitting these species, 51 bryozoan species were previously known in Galicia between 100 and 200 m depth. The 33 species on the whale bone adds 15 additional species to this depth range, bringing the total number to 66 (Table 1). Accordingly, 50% of the species present in this depth range were found on the whale bone. No single small hard substrate has been found to host a similar number of species at this depth. This high number of bryozoan species (and colonies) is comparable only to that found on shells on shallow detritic bottoms (8–30 m depth) in the Rías of NW Spain, where more than 20 species were reported on a single small rock or shell (Reverter Gil 1995). The continental shelf at this depth (128 m) is formed mainly by soft sediment (Rey et al. 2014), which is suboptimal for colonization by bryozoans. Larvae of shallow-water bryozoans can reach this depth but the absence of suitable substrate prevents their survival; the same holds true for the larvae of deeper species. When, however, a rare appropriate substrate appears, it can attract a high diversity of species. This helps explaining the presence of species more typical of shallower or even deeper waters. This also supports the theory that whale-falls serve as intermediate habitats, facilitating habitat colonization—in this case of deep hard substrates on the continental slope (see Smith et al. 2015 and references herein). With the moratorium on commercial whaling and increasing conservation efforts for cetaceans, the number of whale-falls can be expected to increase, making this type of substrate more important in the future.

## Conclusions

This is the first report of a bryozoan community colonizing a whale-fall. The high species diversity is noteworthy, with 33 species identified on a substrate under 0.2 m^2^ surface area. No potential whale-fall specialists were detected: all the species are already known in the area, with most of them in the same depth range, although several may be more frequent in shallower or in deeper waters. However, our knowledge of the bryozoan fauna between 100 and 200 m depth is poor because most studies have been conducted in shallow coastal waters or on the deeper continental slope. Although the lack of regular suitable substrates at these depths makes it difficult to detect a high bryozoan diversity, such intermediate depths deserve closer scrutiny.

## Supplementary Information

ESM 1 (DOCX 20 kb)

## Declarations

### Sampling and field studies

All necessary permits for sampling and observational field studies have been obtained by the authors from the competent authorities and are mentioned in the acknowledgements.

### Data availability

All data generated or analyzed during this study are included in this published article (and its supplementary information files).
