# Supplementary material for: Bryozoan diversity on a whale bone: an uncommon substrate from the continental shelf off NW Spain
Source: Mar Biodivers. 2021 May 14;51(3):50. doi: 10.1007/s12526-021-01189-6 (PMC8550466; doi:10.1007/s12526-021-01189-6)
Supplement: Supplementary file 1 — (DOCX 20 kb) [file 12526_2021_1189_MOESM1_ESM.docx]

Supplementary material

**Species reported from the whale bone, with bibliographic references and samples conserved at the MHNUSC**

**CYCLOSTOMATIDA**

***Diplosolen obelium*** (Johnston, 1838)

Several colonies (MHNUSC-Bry 173, 175a: Reverter-Gil & Fernández-Pulpeiro, 2001). (MHNUSC-Bry 411, 418: unpublished).

***Entalophoroecia deflexa*** (Couch, 1842)

Many colonies (Reverter-Gil & Fernández-Pulpeiro, 2001).

***Plagioecia sarniensis*** (Norman, 1864)

Several colonies (Reverter-Gil & Fernández-Pulpeiro, 2001).

**CHEILOSTOMATIDA**

***Aetea longicollis*** Jullien in Jullien & Calvet, 1903

Several colonies (MHNUSC-Bry 418, 509: Reverter-Gil *et al*., 2019a; MHNUSC-Bry 420: Reverter-Gil *et al*., 2019a: fig. 2(a)). (MHNUSC-Bry 689: unpublished).

***Alderina imbellis*** (Hincks, 1860)

Ovicelled colonies (MHNUSC-Bry 689: Reverter-Gil & Fernández-Pulpeiro, 2001). (MHNUSC-Bry 405: unpublished).

***Arthropoma cecilii*** (Audouin, 1826)

Several colonies (Reverter-Gil & Fernández-Pulpeiro, 2001). (MHNUSC-Bry 418, 420, 509: unpublished).

***Callopora dumerilii*** (Audouin, 1826)

Several ovicelled colonies (MHNUSC-Bry 410: Reverter-Gil *et al*., 2019b).

***Chaperiopsis annulus*** (Manzoni, 1870)

Several dead colonies (MHNUSC-Bry 175a: Reverter-Gil & Fernández-Pulpeiro, 2001).

***Cheiloporina circumcincta*** (Neviani, 1896)

Many ovicelled colonies (MHNUSC-Bry 175a: Reverter-Gil & Fernández-Pulpeiro, 1999a: fig. 4D; MHNUSC-Bry 175b, 178: Reverter-Gil & Fernández-Pulpeiro, 1999a). (MHNUSC-Bry 177: Reverter-Gil & Fernández-Pulpeiro, 2001); (MHNUSC-Bry 408, 409, 411, 413, 414, 417, 509: unpublished).

***Chorizopora brongniartii*** (Audouin, 1826)

Several ovicelled colonies (MHNUSC-Bry 408: Reverter-Gil *et al*., 2019b).

***Cribrilaria arrecta*** Bishop & Househam, 1987

Several ovicelled colonies (MHNUSC-Bry 175a: Reverter-Gil & Fernández-Pulpeiro, 2001).

***Cribrilaria venusta*** Canu & Bassler, 1925

Many ovicelled colonies (MHNUSC-Bry 173, 175b: Reverter-Gil & Fernández-Pulpeiro, 2001). (MHNUSC-Bry 407, 409, 412, 417, 421, 509: unpublished).

***Escharella ventricosa*** (Hassall, 1842)

Several colonies (MHNUSC-Bry 177: Reverter-Gil & Fernández-Pulpeiro, 2001). (MHNUSC-Bry 411, 412, 414, 415: unpublished).

***Escharina vulgaris*** (Moll, 1803)

Several ovicelled colonies (MHNUSC-Bry 178: Reverter-Gil & Fernández-Pulpeiro, 2001).

***Fenestrulina asturiasensis*** Álvarez, 1992c

Several colonies (MHNUSC-Bry 404: Reverter-Gil *et al*., 2019a: fig. 4(b); MHNUSC-Bry 518: Reverter-Gil *et al*., 2019a).

***Figularia figularis*** (Johnston, 1847)

Many ovicelled colonies (MHNUSC-Bry 180: Reverter-Gil & Fernández-Pulpeiro, 2001). (MHNUSC-Bry 408: unpublished).

***Haplopoma sciaphilum*** Silén & Harmelin, 1976

Two small ovicelled colonies (MHNUSC-Bry 520: Reverter-Gil *et al*., 2019a: fig. 3(d, e)).

***Hemicyclopora discrepans*** (Jullien in Jullien & Calvet, 1903)

Several ovicelled colonies (MHNUSC-Bry 175a: Reverter-Gil & Fernández-Pulpeiro, 1999b: fig. 4C; MHNUSC-Bry 176: Reverter-Gil & Fernández-Pulpeiro, 1999b: fig. 4A, B).

***Herentia* sp.**

Several ovicelled colonies: *H. hyndmanni* or *H. thalassae* (Reverter-Gil & Fernández-Pulpeiro, 2001 as *Escharina hyndmanni*; Reverter-Gil *et al*., 2019a).

***Hippothoa flagellum*** Manzoni, 1870

Several colonies (Reverter-Gil & Fernández-Pulpeiro, 2001). (MHNUSC-Bry 416, 417: unpublished).

***Microporella ciliata*** (Pallas, 1766)

Several colonies (Reverter-Gil & Fernández-Pulpeiro, 2001). (MHNUSC-Bry 414, 417: unpublished).

***Reptadeonella insidiosa*** (Jullien in Jullien & Calvet, 1903)

Several colonies (MHNUSC-Bry 179, 180: Reverter-Gil & Fernández-Pulpeiro, 2001). (MHNUSC-Bry 406, 415: unpublished).

***Reteporella couchii*** (Hincks, 1878)

Several young colonies (MHNUSC-Bry 403, 410: Reverter-Gil *et al*., 2019a).

***Rhynchozoon bispinosum*** (Johnston, 1847)

Several colonies (Reverter-Gil & Fernández-Pulpeiro, 2001). (MHNUSC-Bry 509: unpublished).

***Schizomavella (Calvetomavella) discoidea*** (Busk, 1859)

An ovicelled colony (MHNUSC-Bry 567: Reverter-Gil *et al*., 2019b: fig. 5A).

***Schizomavella* (*Schizomavella*) *auriculata*** (Hassall, 1842)

One colony (Reverter-Gil & Fernández-Pulpeiro, 2001). (MHNUSC-Bry 416: unpublished).

***Schizomavella* (*Schizomavella*) *linearis*** (Hassall, 1841)

Many ovicelled colonies (MHNUSC-Bry 173: Reverter-Gil & Fernández-Pulpeiro, 2001). (MHNUSC-Bry 419, 420, 509: unpublished).

***Schizoporella cornualis*** Hayward & Ryland, 1995

Several colonies (MHNUSC-Bry 156b: Reverter-Gil & Fernández-Pulpeiro, 1999a).

***Schizotheca tuberigera*** (Jullien in Jullien & Calvet, 1903)

Several ovicelled colonies (MHNUSC-Bry 179: Reverter-Gil & Fernández-Pulpeiro, 2001, 2007a).

***Scrupocellaria inermis*** Norman, 1868

A small colony (Reverter-Gil & Fernández-Pulpeiro, 2001).

***Smittoidea reticulata*** (J. MacGillivray, 1842)

Several ovicelled colonies (MHNUSC-Bry 415: Reverter-Gil *et al*., 2019b).

***Stephanollona*** ***armata*** (Hincks, 1862)

Two colonies (MHNUSC-Bry 174: Reverter-Gil & Fernández-Pulpeiro, 1999a: figs. 4 E, F; MHNUSC-Bry 178: Reverter-Gil & Fernández-Pulpeiro, 1999a).

***Steraechmella buski*** Lagaaij, 1952

Many colonies (MHNUSC-Bry 173: Reverter-Gil & Fernández-Pulpeiro, 1999a: fig. 1B; MHNUSC-Bry 175b: Reverter-Gil & Fernández-Pulpeiro, 1999a). (MHNUSC-Bry 408, 519: unpublished).

**References**

Bishop JDD, Househam BC (1987) Puellina (Bryozoa; Cheilostomatida; Cribrilinidae) from British and adjacent waters. Bull Br Mus Nat Hist Zool 53:1–63

Canu F, Bassler RS (1925) Les Bryozoaires du Maroc et de Mauritanie. Mem Soc Sci Nat Maroc 10:1–79

Couch RQ (1842) Observations on the sponges of Cornwall. Ann Rep Roy Cornwall Polytechnic Soc 10:41–62

Hincks T (1860) Descriptions of new Polyzoa from Ireland. Q J Microsc Sci 8:275–280

Hincks T (1862) Catalogue of the Zoophytes of South Devon and Cornwall. Ann Mag Natl Hist (Series 3) 9:200–207

Hincks T (1878) Notes on the genus Retepora, with descriptions of new species. Ann Mag Nat Hist (Series 5) 1:353–365

Johnston G (1838) A history of the British zoophytes. W.H. Lizars, Edinburgh

MacGillivray J (1842) Catalogue of the marine zoophytes of the neighbourhood of Aberdeen. Ann Mag Nat Hist (Series 1) 9:462–469

Moll JPC (1803) Eschara, ex zoophytorum, seu, phytozoorum ordine pulcherrimum ac notatu dignissimum genus, novis speciebus auctum, methodice descriptum et

iconibus ad naturam delineatis illustratum. Camesiniana, Vindobonae

Norman AM (1868) Notes on some rare British Polyzoa, with descriptions of new species. Q J Micros Sci 8:212–222
